# Diagnosis of Chronic Pulmonary Aspergillosis: Challenges and Limitations Response to “Diagnosis of Chronic Pulmonary Aspergillosis: Which Is the Best Investigation?” and “Diagnosis of Chronic Pulmonary Aspergillosis”

**DOI:** 10.4269/ajtmh.23-0053c

**Published:** 2023-04-17

**Authors:** Vítor Falcão De Oliveira, Marcello Mihailenko Chaves Magri

**Affiliations:** Department of Infectious and Parasitic Diseases Hospital das Clínicas University of São Paulo São Paulo, Brazil E-mail: vitorfalcaodeoliveira@gmail.com; Department of Infectious and Parasitic Diseases Hospital das Clínicas University of São Paulo São Paulo, Brazil E-mail: marcello.magri@hc.fm.usp.br

Dear Editor,

We are grateful for the interest in our recently published article on the diagnosis of chronic pulmonary aspergillosis (CPA).^1^ We feel that it is important to discuss the challenges and limitations of diagnosis, mainly in low- and middle-income countries.^2^ This is especially important because diagnostic assays are not available in limited resource countries, and there is a gap in knowledge on diagnostic aspects, such as the cutoff for galactomannan (GM).

Serology by immunoprecipitation methods (immunodiffusion or counterimmunoelectrophoresis) may be considered inferior to serum *Aspergillus fumigatus*-specific IgG for CPA, but immunoprecipitation tests are still the main available tests in many countries, including Brazil.^3^ The current use of precipitation tests can be explained by the low cost of this technique and its greater availability.^3^ Serology is the best non-invasive assay for diagnosis,^4^^,^^5^ even if performed by immunoprecipitation, with a positivity rate of 81%, and it was the best assay for diagnosis in our study.^1^

The updated European Society for Clinical Microbiology and Infectious Diseases and European Respiratory Society guidelines for the diagnosis of CPA are recommended, with modifications in resource-constrained settings.^6^^,^^7^ We disagree that histology is the gold standard for diagnosing CPA, as it may not be positive in all cases, especially for specimens that are collected blindly and may not be obtained from the preferred site.^8^ Our positivity rate of histology was 78%, reinforcing the limited sensitivity of this assay for diagnosing CPA.

Several studies that have evaluated GM cutoffs, with variable results, and variable sensitivity and specificity.^9^^–^^15^ Thus, a clear cutoff value has not been established for CPA.^11^ In addition, the measurement of GM in bronchoalveolar lavage fluid is challenging in resource-limited settings given that bronchoscopy is not routinely performed.^16^

We agree with the limitations highlighted by Bongomin and colleagues. Despite the limitations, our results can inspire other studies. Diagnosing CPA in low- and middle-income countries is challenging, justifying studies that may identify simpler alternatives to invasive and/or expensive tests.
